# Study of a Multicriterion Decision-Making Approach to the MQL Turning of AISI 304 Steel Using Hybrid Nanocutting Fluid

**DOI:** 10.3390/ma14237207

**Published:** 2021-11-26

**Authors:** Vineet Dubey, Anuj Kumar Sharma, Prameet Vats, Danil Yurievich Pimenov, Khaled Giasin, Daniel Chuchala

**Affiliations:** 1 Centre for Advanced Studies, Dr. A.P.J Abdul Kalam Technical University, Lucknow 226031, India; sharmaanuj79@gmail.com (A.K.S.); prameetvats@gmail.com (P.V.); 2 Department of Automated Mechanical Engineering, South Ural State University, Lenin Prosp. 76, 454080 Chelyabinsk, Russia; danil_u@rambler.ru; 3 School of Mechanical and Design Engineering, University of Portsmouth, Portsmouth PO1 3DJ, UK; Khaled.giasin@port.ac.uk; 4 Faculty of Mechanical Engineering and Ship Technology, Gdańsk University of Technology, 80-233 Gdańsk, Poland; daniel.chuchala@pg.edu.pl

**Keywords:** machining, turning, AISI 304 steel, minimum quantity lubrication (MQL), temperature, lubrication, nanofluids, optimization, wear

## Abstract

The enormous use of cutting fluid in machining leads to an increase in machining costs, along with different health hazards. Cutting fluid can be used efficiently using the MQL (minimum quantity lubrication) method, which aids in improving the machining performance. This paper contains multiple responses, namely, force, surface roughness, and temperature, so there arises a need for a multicriteria optimization technique. Therefore, in this paper, multiobjective optimization based on ratio analysis (MOORA), VIseKriterijumska Optimizacija I Kompromisno Resenje (VIKOR), and technique for order of preference by similarity to ideal solution (TOPSIS) are used to solve different multiobjective problems, and response surface methodology is also used for optimization and to validate the results obtained by multicriterion decision-making technique (MCDM) techniques. The design of the experiment is based on the Box–Behnken technique, which used four input parameters: feed rate, depth of cut, cutting speed, and nanofluid concentration, respectively. The experiments were performed on AISI 304 steel in turning with minimum quantity lubrication (MQL) and found that the use of hybrid nanofluid (Alumina–Graphene) reduces response parameters by approximately 13% in forces, 31% in surface roughness, and 14% in temperature, as compared to Alumina nanofluid. The response parameters are analyzed using analysis of variance (ANOVA), where the depth of cut and feed rate showed a major impact on response parameters. After using all three MCDM techniques, it was found that, at fixed weight factor with each MCDM technique, a similar process parameter was achieved (velocity of 90 m/min, feed of 0.08 mm/min, depth of cut of 0.6 mm, and nanoparticle concentration of 1.5%, respectively) for optimum response. The above stated multicriterion techniques employed in this work aid decision makers in selecting optimum parameters depending upon the desired targets. Thus, this work is a novel approach to studying the effectiveness of hybrid nanofluids in the machining of AISI 304 steel using MCDM techniques.

## 1. Introduction

Machining is a material removal process, in which undesired material is removed from the workpiece to give it a final shape. Different machining operations, such as turning, milling, grinding, and drilling, etc., are used in the manufacturing industry for metal cutting processes. The machining process aims to provide dimensional accuracy to the workpiece. Turning is one of the most widely used metal removal processes, used generally for cylindrical parts. To attain enhanced productivity, the wear of the tool and the obtained surface roughness of the workpiece must be minimal. At the interface of the cutting tool and workpiece, a large amount of heat is generated because of friction. This heat results in temperature generation, affecting tool life and the surface quality of the workpiece. Among the different varieties of steel alloys, the turning of AISI 304 steel is widely used in industries because of its diverse applications. There are a few challenges in the machining of AISI 304 steel alloy, as it possesses lower thermal conductivity along with the tendency of work hardening [1]. Thus, while machining AISI 304 steel, issues of rapid tool wear and increased cutting force are encountered, along with an increased cutting temperature [2]. For overcoming this temperature, cutting fluid is applied at the machining zone. The traditional approach to the application of cutting fluid is effective, but when used to an excess degree, it can cause a detrimental effect on human health as well as the environment. 

To limit the use of traditional cutting fluid, the novel hybrid technique of minimum quantity lubrication (MQL) can be employed in the vicinity of the machining zone [3]. In this technique, the cutting fluid is engaged in the form of a spray, by applying pressurized air [4]. Hegab and Kishawy [5] used alumina and multiwalled carbon nanotube to investigate their effect on the energy consumption and the surface finish generated in the MQL assisted turning of Inconel 718. The carbon nanotube gave a better result than alumina and it was revealed that the weight % of the nanoparticle had a significant effect on the response parameters. The enhanced tribological and heat transfer properties of the nanoparticles added in the cutting fluid led to the improvement in surface characteristics by improving the interface bond between the Inconel surface and the cutting tool used. Sen et al. [6] performed a milling operation using a hybrid mixture of palm and castor oil with a mist lubrication technique. The reduction in surface roughness, by 16.14%, and 7.97% reduction in specific cutting energy, is reported. Duc et al. [7] performed hard turning on 90CrSi steel with minimum quantity lubrication. Alumina and molybdenum disulphide nanofluids are utilized for cutting fluid. A reduction in cutting force with an increase in thrust force is reported using MoS_2_ nanofluid. The use of both the nanoparticles in the MQL technique led to the improved performance of the carbide insert, due to the rise in the property of the base fluid in terms of thermal conductivity and lubrication. Bai et al. [8] studied the effect of different fluids using the minimum quantity lubrication technique on the response parameters. As per the authors, MQL or near dry machining is a suitable alternative for flood cooling in reducing environmental hazards, as well as production costs. The use of nanofluids as a coolant is seen as an emerging concept for machining purposes, as they possess enhanced heat transfer capabilities [9]. Do and Hsu [10] performed machining on AISI H13 and analysed the surface roughness using MQL. Higher cutting speed and low depth of cut resulted in improved surface finish using MQL. Dubey et al. [11] reviewed different methods of temperature measurement while machining. Prediction of temperature using thermocouples was found to be suitable. In another work, Dubey et al. [12] studied the effect of different cooling mechanisms on turning. Among various techniques, MQL was reported to be the most efficient lubrication method. Gupta et al. [13] optimized machining parameters in the turning of titanium alloy under the mist lubrication technique. The result revealed lower cutting force, using graphite nanofluids as it formed lower droplets because of a lower viscosity than the other two nanofluids, and resulted in deeper penetration at the machining zone. In the case of tool wear, graphite nanofluids also outperformed, as they possess better thermal conductivity than the other two and aided in dissipating heat and retaining the cutting tool hardness. Saini et al. [14] experimented on AISI-4340 steel under MQL conditions using different carbide inserts. The application of MQL resulted in a decrease in temperature of the chip–tool interface, thus maintaining the sharpness of the cutting edges of the tool. Singh et al. [15] investigated surface finish, cutting force, and tool wear on the turning of titanium alloy. The results revealed an enhancement in surface finish, by 15%, and a reduction in cutting force by using the near dry machining technique. Qu et al. [16] studied the machining of a ceramic matrix composite, with dry, flood, and minimum quantity lubrication. The improved surface finish obtained using nanofluids assisted MQL, along with less consumption of the cutting fluid in comparison to other lubrication techniques.

With the advancement in studies of nanofluids as lubricants in machining operation, researchers are now focussing on using hybrid nanofluids for enhanced heat transfer characteristics [17]. Babar and Ali [18] reviewed the synthesis and thermophysical properties of hybrid nanofluids. It was suggested that hybrid nanofluids possess superior thermal characteristics over mono nanofluids because mono nanofluid forms clusters, thus increasing the diameter of the particles and, thus, leading to an increase in pumping power and viscosity. The thermophysical characteristic of nanofluids (viscosity, specific heat, viscosity, and density) is improved by enhancing the nanoparticle concentration. Kumar et al. [19] studied the tribological behaviour of nanofluid on different categories of steel. It was revealed that the introduction of nanofluid aided in minimizing wear. Jamil et al. [20] used combinations of alumina and carbon nanotube particles for the hybrid nanofluids machining of titanium alloy with MQL. The obtained result was compared with cryogenic cooling and an improvement in tool life by 23% was observed. A reduction of 11.8% was suggested by the authors in cutting temperature using cryogenic cooling, in comparison to MQL. Zhang et al. [21] compared the effect of hybrid nanofluid with single nanofluid on response parameters while machining on nickel alloy. The application of alumina and silicon carbide hybrid nanofluids resulted in a reduction of cutting forces and surface roughness, respectively, as both the nanofluids gave a synergistic effect and improved the grinding performance. Gugulothu and Pasam [22] investigated the performance of carbon nanotube and molybdenum disulphide nanoparticle enriched cutting fluid for turning 1040 steel. An increase in thermal conductivity is noticed by increasing the particle size, while a decrease in viscosity is encountered when rising in temperature. A reduction in surface roughness, by 28.53% and 18.3%, is reported when compared with dry machining and traditional cutting fluid. Kumar et al. [23] performed machining on silicon nitride and compared the result with mono and hybrid nanofluids. The cutting force and surface roughness were reduced by 27% and 41%. Abbas et al. [24] optimized the turning parameters using Edgeworth–Pareto method for achieving minimum turning time. The obtained surface finish reported is 0.8µm. In another study, Abbas et al. [25] performed a sustainability assessment related to power consumption and surface characteristics in the machining of AISI 1045 steel. The use of alumina nanoparticles in mist lubrication significantly improved the surface characteristic and minimized the power consumption. The effect on response parameters can be attributed to the alumina nanofluid’s spraying ability, enhanced sliding behaviour, less friction, and seizure characteristic at the tool–workpiece contact. Alajmi and Almeshal [26] used artificial intelligence to optimize surface roughness in the turning of AISI 304 steel. It was revealed that ANFIS-QPSO resulted in a more accurate prediction of surface roughness. Su et al. [27] a used multiobjective criterion for optimising machining parameters of AISI 304 steel. The reduction in surface roughness and specific energy consumption was reported to be 66.90% and 81.46%. Khan et al. [28] performed a grinding operation on D2 steel using an alumina wheel, and compared dry machining with MQL grinding. The effectiveness of heat dissipation and the penetration property of the cutting fluid using MQL gave better results. Li et al. [29] investigated tool wear and surface topography in the turning of austenitic steel. Response surface methodology was used as the optimization technique. The effective cutting parameters obtained were 120 mm/min cutting speed and 0.18 feed rate along with 0.42 mm depth of cut. 

From the literature, it is evident that the machining of AISI 304 steel has been attempted by different researchers using nanofluids in improving the machining performances in terms of reduced cutting force, tooltip temperature, and surface roughness. The optimization of the process parameters is performed using Taguchi, grey relational analysis, genetic algorithm, and response surface methodology, but very little work is reported on an analysis of optimal parameters using multicriterion decision making (MCDM) techniques using minimum quantity lubrication. In the present work, alumina and graphene nanoparticles are hybridized in different volumetric concentrations. The performance of the hybrid nanofluids is analyzed in terms of cutting forces, surface roughness, and nodal temperature for the MQL turning of AISI 304 steel. The study aims to analyze the synergistic effect of the hybrid nanofluids on the response parameters for the MQL turning of steel, and suggest the optimum parameter and cutting fluid that can be used by researchers and industries while machining steel. The results obtained are further compared with that of alumina particle nanofluid. Furthermore, the selection of the optimized machining level parameter and their respective ranking is ascertained using three MCDM techniques, namely, MOORA (Multiobjective Optimization Method by Ratio Analysis), VIKOR (VlseKriterijumska Optimizacija I Kompromisno Resenje), and TOPSIS (technique for order performance by similarity to ideal solution).

## 2. Materials and Methods

**Figure 1:**
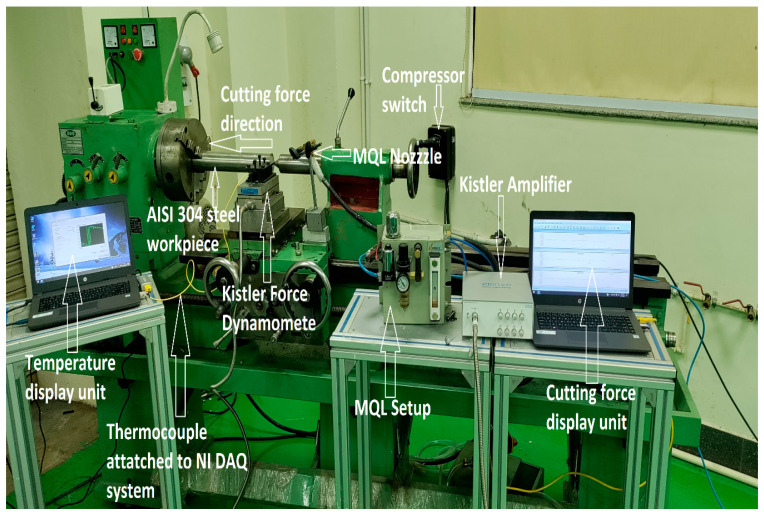
Experimental setup for MQL turning of AISI304 steel.

The experiment was performed using a conventional lathe Duo machine (Duo Machine Corps, Rajkot, India). Turning was carried on an AISI 304 steel workpiece of 60 mm diameter, whose chemical composition is mentioned in Table 1. WIDIA’s tungsten carbide inserts (CNMG 120408) of grade TN 2000 and corner radius of 0.8 were used as a cutting tool material which is clamped mechanically on WIDIA’s tool holder. The experimental response, such as cutting force, was measured by using a piezoelectric Kistler dynamometer (9257B). It consists of a charge amplifier of Type 5697A1, comprising hardware for the data acquisition and the DynoWare software (3.1.2.0) for operating and storing the value of average cutting force. Turning operation was performed for 250 mm length of cylindrical workpiece and the average value of cutting force was recorded. Mitutoyo surface roughness tester (SJ210) was used for average surface roughness measurement (R_a_). It consists of a probe comprising of the diamond tip of a 2 µm radius that traverses on the workpiece. The cut off length is 0.08 mm and measuring speed is 0.25 mm/s, and the retraction speed of the probe is 1mm/s. The temperature measurement was performed using a K-type thermocouple, whose one end is clamped in a carbide insert, while the other end is attached to National Instrument’s data acquisition system, which recorded the cutting temperature. The cutting fluid used for machining is biodegradable oil based, which is enriched with water based alumina nanofluid and alumina–graphene hybrid nanofluid. The selection of nanofluids is carried out to analyze the synergistic effect of alumina (high conductivity) and graphene (high thermal conductivity along with lubricity) on turning in MQL environment. The combined properties of both nanoparticles are essential for any cutting fluid used in machining. The samples of mono and hybrid nanofluids were prepared in a volumetric ratio of 90:10 in three varying volumetric concentrations of 0.5%, 1%, and 1.5%, respectively. For the discharge of the cutting fluid, a minimum quantity lubrication setup was used. The experiments were repeated thrice and the average value was taken of the responses for better accuracy. The experimental setup is shown in Figure 1.

Design of experiment is made by using MINITAB-19 and for statistical analysis response surface methodology’s Box–Behnken design was used, with four factors at three different levels where the factors are, namely, depth of cut, feed rate, cutting velocity, and nanofluid concentration, which is shown in Table 2. Due to 4 factors with 3 levels, the design contains 27 possible combinations to perform experiments. Table 3 contains all 27 combinations which give the most effective results of response parameters.

Optimization is very important in a production system because it helps to achieve good product quality at minimum cost. In this paper, there are three response parameters, and optimizing them individually may take a significant amount of time, effort and increase process complexity. Therefore, this paper deals with four optimization techniques to obtain a better result.

### 2.1. Response Surface Methodology

Response surface methodology is a collection of statistical and mathematical techniques which are useful for the modeling and analysis of a problem in which the response of interest is influenced by multiple variables and the objective is to optimize the response. Response surface methodology is used for surface analysis of response parameters; along with that, problems formulation and process optimization can also be performed using RSM [30]. 

### 2.2. Multicriterion Decision Making

**Figure 2:**
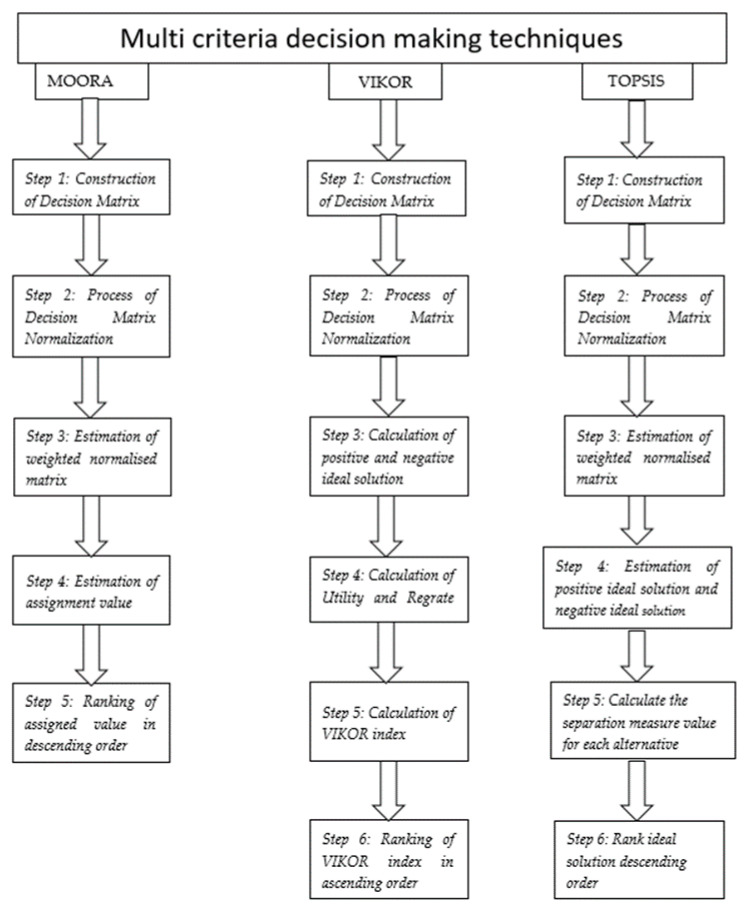
Methodology of different MCDM techniques.

Multicriteria decision making is mainly aimed at the optimization of conflicting responses, but in this paper, it is utilized for optimizing multiple criteria of nonconflicting nature. It is very useful when the number of response parameters is large in the count, because it calculates the optimized results for two responses and more than two responses in the same number of steps. The methodology used in these techniques is shown in Figure 2. Here, the goal is to mainly check the reliability of three MCDM techniques (MOORA, VIKOR, TOSIS) for nonconflicting responses [31,32].

#### 2.2.1. Multiobjective Optimization Based on Ratio Analysis (MOORA)

MOORA is a simpler and popular MCDM technique; it is used to simultaneously optimize two or more than two conflicting/nonconflicting response parameters [33,34]. It is mainly used for the quantitative attribute.

#### 2.2.2. VIKOR

The VIKOR method is a multicriteria decision making (MCDM) or multicriteria decision analysis method. It was originally developed by Serafim Opricovic (1979-80) to solve decision problems with conflicting and noncommensurable (different units) criteria. It is used to simultaneously optimize two or more two responses. The decision maker desires to have a solution that is nearest to the ideal, whereas the alternatives are evaluated as per the established criteria. VIKOR ranks alternatives and determines the solution, named compromise, that is the closest to the ideal [35].

#### 2.2.3. TOPSIS

TOPSIS is an MCDM technique. It is also used to calculate the optimized value when responses are large in number. It is a technique for order of preference by similarity to the ideal solution. It was developed by Ching-Lai Hwang and Yoon in 1981 and, further, it was developed by Yoon in 1981 and Harang in 1993 [36].

## 3. Results

In this paper, there are three major responses, namely, force, surface roughness, and temperature. All the three selected response parameters come under the nonbeneficial category; therefore, all of them should be at their minimum. To minimize them, proper lubrication and cooling are required at the machining interface. Therefore, in the present paper, mono and hybrid nanofluids with an MQL setup is used for cooling and lubrication purpose. As per the experimental results, it is found that the response parameters give more promising results, as they aided in reducing cutting forces, tool temperature, and surface roughness with hybrid nanofluids, as compared to single nanofluids alone, as shown in Table 4. 

### 3.1. Response Surface Methodology 

RSM is used as a multipurpose technique: it can help to create mathematical model to predict the response and it can also help to analyze the surface response through the response surface curve for better understanding the effect of a process parameter on a response parameter; it also helps in the analysis of variance of process parameters and it can also calculate the optimized parameter. In this paper, a second degree model is used for performing data analysis and to determine the significance of the model’s parameters, calculation of mean response, and to arrive at optimum operating conditions on the control variables that helps to achieve a maximum or a minimum response over a certain region of interest. Therefore, after getting response parameters (Table 4), the quadratic model has been developed for the analysis of variance to check the stability and significance of the response, as well as process parameters [37]. The mathematical model for response parameters is discussed in the equations given below:

For alumina

Cutting Force = −370 − 1.86v_c_ + 3378 f_o_ + 874 a_p_ − 49 np% − 0.0055 v_c_ * v_c_ − 13159 f_o_ * f_o_ − 418 a_p_ * a_p_ − 12.0 np%*np% + 5.5 v_c_ * f_o_ + 2.13 v_c_ * a_p_ − 0.067 v_c_ *np%+ 931 f_o_ * a_p_ + 354 f_o_ *np% − 13.9a_p_*np%. (1)

Surface Roughness = 2.34 + 0.0069 v_c_ − 23.52 f_o_ + 1.68 a_p_ − 0.306 np% − 0.000001 v_c_ * v_c_ + 143.7 f_o_ * f_o_ − 0.169 a_p_ * a_p_ − 0.138 np%*np% − 0.0275 v_c_ * f_o_ − 0.00796 v_c_ * a_p_ − 0.00317 v_c_ *np%− 4.02 f_o_ * a_p_ + 6.38 f_o_ *np% − 0.234 a_p_ *np% (2)

Temperature = −95 − 2.01 v_c_ + 2248 f_o_ + 433 a_p_ − 108.6 np%− 0.00094 v_c_ * v_c_ − 3501 f_o_ * f_o_ − 212.1 a_p_ * a_p_ − 16.6 np%*np% − 4.79 v_c_ * f_o_ + 1.896 v_c_ * a_p_ + 1.357 v_c_ *np% − 532 f_o_ * a_p_ + 153 f_o_ *np% − 17.5 a_p_ *np% (3)

For alumina–graphene

Cutting Force = −350 − 2.27 v_c_ + 5998 f_o_ + 646 a_p_ − 146 np%+ 0.0044 v_c_ * v_c_ − 22338 f_o_ * f_o_ − 351 a_p_ * a_p_ − 7.6 np%*np% − 7.6 v_c_ * f_o_ + 1.76 v_c_ * a_p_ + 0.32 v_c_ *np% + 1223 f_o_ * a_p_ + 292 f_o_ *np%+ 59 a_p_ *np% (4)

Surface Roughness = 1.20 + 0.01201 v_c_ − 14.02 f_o_ + 0.988 a_p_ − 0.023 np% − 0.000020 v_c_ * v_c_ + 97.6 f_o_ * f_o_ − 0.038 a_p_ * a_p_ − 0.120 np%*np% − 0.0399 v_c_ * f_o_ − 0.00541 v_c_ * a_p_ − 0.00415 v_c_ *np% − 2.75 f_o_ * a_p_ + 4.39 f_o_ *np%− 0.143 a_p_ *np% (5)

Temperature = 47 − 3.66 v_c_ + 1187 Feed + 399 a_p_ − 134.9 np%+ 0.00684 v_c_ * v_c_ − 4758 Feed*Feed − 171.3 a_p_ * a_p_ − 26.7 np%*np% + 4.54 v_c_ *Feed + 1.171 v_c_ * a_p_ + 1.230 v_c_ *np%− 459 Feed* a_p_ + 615 Feed*np% − 15.2 a_p_ *np% (6)

The above mentioned regression model helps to predict the response parameters, i.e., cutting force, temperature, roughness. Now, the analysis of variance is required to analyze the significance and influence of the process parameters and their factors on response parameters. ANOVA was carried out at a 95% confidence level, which means the *p*-value of the factors must be less than 0.05 to satisfy the condition of a significant factor criteria. The coefficient of determinant, i.e., R^2^ and adjusted R^2^, is also one of the parameters to show the significance of experimental results. A regression model helps to calculate the coefficient of the determinant, and it should be more the 80% because, for the experimental results, 80% is an acceptable limit [38]. The ANOVA analysis, describing the *p*-value and percentage contribution of the response parameters in alumina and alumina–graphene enriched cutting fluid, is given in Table 5 and Table 6.

In Table A1, the analysis of the variance for force has been carried out to analyze the significance of the process parameters and their impact on the response parameter i.e., force. Table A1 signifies that depth of cut has a major impression on cutting force, approximately 65.2841%, which is the highest among all of the process parameters and their factors. As discussed above, parameters having a p-value <0.05 are significant; therefore, velocity, feed, depth of cut, np% and velocity*velocity, velocity* depth of cut, feed*np% are the significant parameters for cutting force. The coefficient of determinant is also used to show the significance and accuracy of experimental results: if R^2^ and adjusted R^2^ is greater than 90% the output is acceptable. In the case of cutting force, R^2^ is 96.25% and adjusted R^2^ is 91.87%. In Table A2, the analysis of the variance for roughness has been carried out, to analyze the significance of the process parameters and their impact on response parameter i.e., surface roughness. Table 5 signifies that the feed rate makes a major impression on surface roughness, approximately 62.38%, which is the highest among all of the process parameters and their factors. The coefficient of determinant is also used to show the significance and accuracy of experimental results: if R^2^ and adjusted R^2^ is greater than 90% the output is acceptable. In the case of surface roughness, R^2^ is 95.76% and adjusted R^2^ is 90.81%. In Table A3, ANOVA signifies that depth of cut makes a major impression on tool temperature, approximately 55.53%, which is the highest among all of the process parameters and their factors. The coefficient of determinant also use to show the significance and accuracy of experimental results, so, in the case of tool temperature, R^2^ is 95.52% and adjusted R^2^ is 90.29%.

In Table A4, an analysis of the variance for cutting force has been carried out to analyze the significance of the process parameter and their impact on cutting force. Table A4 signifies that depth of cut makes a major impression on cutting force, it contributes approximately 68.977% which is the highest among all the process parameters and their factors. The coefficient of determinant is also used to show the significance and accuracy of experimental results: if R^2^ and adjusted R^2^ is greater than 90% the output is acceptable. In the case of surface roughness, R^2^ is 94.96% and adjusted R^2^ is 89.09%. In the case of Table A5, the feed rate shows the major impact on surface roughness. It contributes approximately 57.547%, which is the highest among all the process parameters and their factors; while the coefficient of the determinant of experimental calculated R^2^ as 95.60% and adjusted R^2^ as 90.47%. In Table A6, ANOVA signifies that the depth of cut makes a major impression on tool temperature, approximately 48.52%, which is the highest among all of the process parameters and their factors. The coefficient of determinant is also used to show the significance and accuracy of experimental results, so, in the case of tool temperature, R^2^ is 93.53%and adjusted R^2^ is 85.99%%.

**Figure 3:**
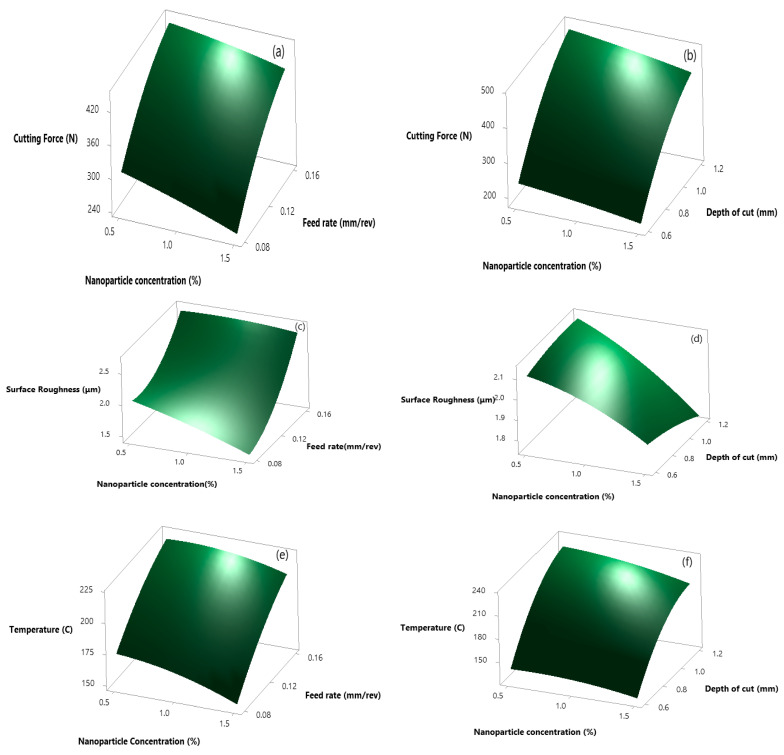
Response surface plot for alumina nanofluid for cutting force. (**a**) np% Vs f_o_; (**b**) np% Vs a_p_; for surface roughness (**c**) np% Vs f_o_; (**d**) np% Vs a_p_ and for cutting temperature (**e**) np% Vs f_o_; (**f**) np% Vs a_p_.

As ANOVA signifies the impact of process parameters on response parameters, similarly, the response surface curve shows the variation in response parameters by varying input. Figure 3 represents the response surface curve at variable feed, depth of cut, and nanofluid concentration for Al_2_O_3_ nanoparticles. Figure 3a,b shows variation in forces, with 0.08 feed rate, 1.5% nanofluid concentration and 0.6 depth of cut force as minimum. The reduction in cutting force can be attributed to the rolling effect produced by the spherical size of alumina, which possesses high strength, hardness and delivers enough abrasive resistance in the process of friction and aids in minimizing the frictional coefficient in the zone of contact [39]. Figure 3c,d explains variation in surface roughness, at maximum nanofluid concentration and minimum feed rate, depth of cut surface roughness is minimum as alumina resulted in minimizing the adhesion between the tool insert and workpiece and forming a tribo film, thus resulting in improved surface quality [40]. Similarly, Figure 3e,f, shows the responses plot for temperature, and, in both cases, at maximum nanofluid concentration and minimum feed rate responses are minimum.

**Figure 4:**
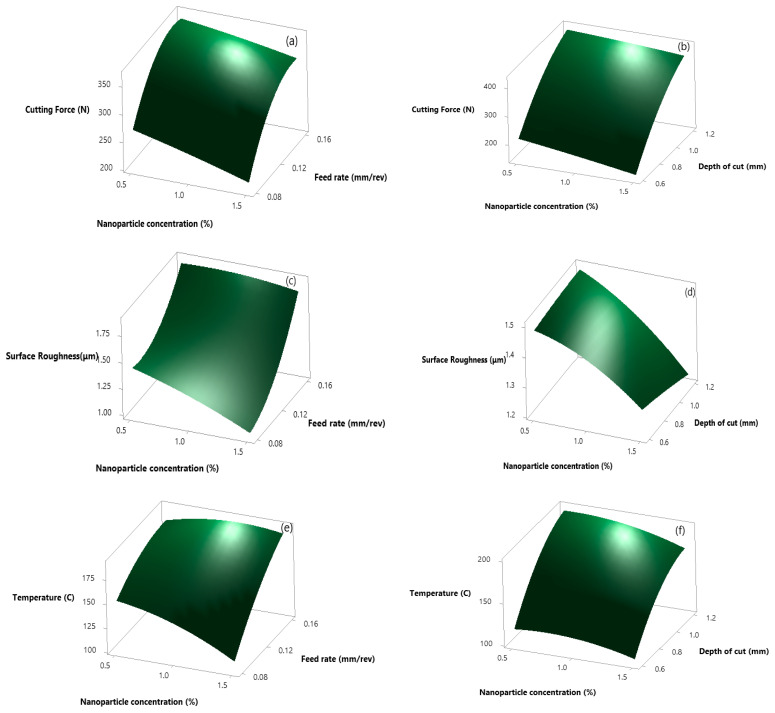
Response surface plot for alumina-graphene nanofluids for cutting force. (**a**) np% Vs f_o_; (**b**) np% Vs a_p_; for surface roughness (**c**) np% Vs f_o_; (**d**) np% Vs a_p_ and for cutting temperature (**e**) np% Vs f_o_; (**f**) np% Vs a_p_.

As discussed in the case of alumina nanofluid, similar results are shown in the case of hybrid nanofluid (alumina–graphene). Figure 4 shows the variation in responses (force, surface roughness, and temperature) by varying input parameters. Figure 4a, b shows the response surface curve for cutting force, at the minimum value of feed rate, depth of cut, and maximum nanofluid concentration. The reduction in cutting force is more in the case of alumina–graphene hybrid nanofluids machining as compared to alumina nanofluids due to the exfoliation of the sheet like structure of graphene because of the shearing action produced by the chip on the tool rake face. In Figure 4c–f, surface roughness and temperature, at a 0.08 feed rate, 1.5% nanofluid concentration, and 0.6 depth of cut force is minimum. After analyzing both the figures, force, surface roughness, and temperature increase, while the increase in depth of cut and feed at minimum nanofluid concentration and decreases with a decrease in depth of cut and feed at maximum concentration [37].

### 3.2. MOORA Analysis for Mono and Hybrid Nanofluid

MOORA is used for selecting the best optimum parameters. Table A7 and Table A8 contain the decision matrix, normalized decision matrix, and assessed value for alumina and alumina–graphene based nanofluid results. The decision matrix contains all the response parameters, such as force, surface roughness, and temperature. Normalization of the matrix is performed to convert them into dimensionless quantities. After normalization of the decision matrix, it will be further multiplied with the weight factor and convert the matrix into the weighted normalized matrix; after that, assessment values (B_i_) for the considered alternatives were determined and ranking them in descending order, the maximum value is ranked as the best (rank 1) and the minimum is ranked as the worst (rank27) [41,42]. The combined analysis of different MCDM techniques and the respective ranks obtained from the decision-making criteria used in mono and hybrid nanofluid cutting fluid based machining is mentioned in Table 7 and Table 8.

### 3.3. VIKOR Analysis for Mono and Hybrid Nanofluid

VIKOR is a multicriteria optimization technique used for selecting the best optimum parameters in a conflicting and nonconflicting response. Table A9 and Table A10 contain the decision matrix, normalized decision matrix, and VIKOR index for alumina and alumina–graphene based nanofluid results. The decision matrix contains all the response parameters, such as force, surface roughness, and temperature. Normalization of the matrix is performed to convert them into dimensionless quantities. After normalization of the decision matrix, it will be further multiplied with the weight factor and convert the matrix into the weighted normalized matrix, at the end, the VIKOR index was determined and they were ranked in ascending order: the minimum VIKOR index value is ranked as the best (rank 1) and the maximum VIKOR index is ranked as the worst (rank27) [43,44,45].

### 3.4. TOPSIS Analysis for Mono and Hybrid Nanofluid

TOPSIS analysis is used to predict ideal solutions in multiresponse parameters. Table A11 and Table A12 contain the decision matrix, normalized decision matrix, and relative ideal solution for alumina and alumina–graphene based nanofluid results. Decision matrices contain response parameters such as force, roughness, and temperature. After forming a decision matrix, normalization of the matrix is required to convert them into dimensionless quantities. Afterward, the weighted normalized matrix has been formed by multiplying the weight factor with the normalized matrix. Next, the positive ideal solution (s+) and negative ideal solutions (s−) were calculated. Ranking of the ideal solution has been assigned by arranging them in descending order [46,47,48,49].

The optimum results obtained from all four techniques are summarized in Table 9. In all four techniques, RSM gives the minimum optimized results, whereas the rest of the three techniques give similar optimum results. RSM gives the optimum output value for the new input parameters, which are different from the input parameters mentioned in the design of the experiment; whereas the MCDM techniques give ideal results from the 27 experimentals used in this paper [50,51]. 

## 4. Conclusions

The methodology used in this paper, of using multicriterion decision-making techniques in selecting the optimum parameters while performing turning operations with mono and hybrid nanofluids enriched with cutting fluid, is novel in this field. As nanofluids are very costly, their use in an efficient manner needs to be studied. The present study can help researchers and industries in choosing the optimum parameters while machining AISI 304 steel, which has wide applications. As per the experimental results, hybrid nanofluids seem to be more effective than a single nanofluid. This paper deals with three response parameters—force, surface roughness, and temperature—all of which are nonbeneficial; therefore, they should have the minimum value. After comparing the results, the following conclusions are made and summarized below:

- The use of hybrid nanofluid (alumina–graphene) resulted in an average reduction of response parameters by approximately 13% in cutting forces, 31% in surface roughness, and 14% in temperature, when compared to alumina nanofluid.
- It can be seen that the use of nanoparticle concentration in a lesser amount resulted in better surface characteristics and resulted in the lowering of cutting forces.
- Analysis of variance revealed the influence of input parameters on the response parameters. In both the cases, i.e., single and hybrid nanofluid, depth of cut showed a major impact while calculating force and temperature. The contribution of the depth of cut is approximately 65.81% and 57.63% in the case of single nanofluid while in the case of hybrid the % contributions are 68.38% and 51.14%, respectively. However, in the case of surface roughness, the most influenced parameter is the feed rate: its contributions in the cases of single and hybrid nanofluid are 63.18% and 58.47%, respectively.
- Response surface methodology is used for optimizing the response. As per RSM, the best process parameters for optimum response in the case of Al2O3 are 86.667 m/min velocity, 0.08 mm/min feed rate, 0.6 mm depth of cut, and at 1.5% of nanoparticle concentration. In the case of alumina–graphene, the suitable parameters for optimum results are 110.909 m/min velocity, 0.08 mm/min feed rate, 0.6484 mm depth of cut, and a nanoparticle concentration of 1.5%, respectively.
- The multicriteria decision-making techniques are used, such as MOORA, VIKOR, and TOPSIS for nonconflicting, nonbeneficial responses at 0.5 weight factor. According to the MCDM techniques, the best input parameter for optimum response is at 90 m/min velocity, 0.6 mm depth of cut, 0.08 mm/min feed rate, and 1% nanoparticle concentration.
- All three MCDM techniques showed similar responses, at a constant or fixed weight factor of 0.5.

The present paper discusses machining performance using hybrid nanofluids. Here, graphene was used for developing hybrid nanofluids. Though it gave desirable results when compared to alumina, it is costly, so there is a need to find a cheaper alternative for graphene for hybridization, so that machining cost can be minimized. Moreover, in this research, both the nanoparticles (alumina–graphene) were mixed in a fixed mixing ratio of 90:10. There is a need to use different mixing ratios and further optimize the mixing ratio so that the optimum value can be obtained. In the future, further research can be performed on the optimization of MQL parameters. Furthermore, work on the hybridization of MCDM techniques can also be done. The thermal modeling of the cutting tool in multiphase using hybrid nanofluids is yet to be explored.

## Figures and Tables

**Table 1:** Chemical constituents of AISI 304 steel.

Elements	S	P	C	Mo	Cu	Si	Mn	Ni	Cr	Fe
Weight %	0.02	0.027	0.065	0.13	0.14	0.3	1.78	8.1	18.2	71.2

**Table 2:** Input parameters used in the current study.

Levels/Factors	−1	0	1
Depth of cut (mm)	0.6	0.9	1.2
Feed rate (mm/rev)	0.08	0.12	0.16
Cutting speed (m/min)	60	90	120
Nanofluid concentration (wt.%)	0.5	1.0	1.5

**Table 3:** Design of Experiment.

S.No.	Cutting Speed (m/min)	Feed Rate (mm/rev)	Depth of Cut (mm)	Nanoparticle Concentration (%)
1	90	0.16	1.2	1.0
2	60	0.12	1.2	1.0
3	120	0.12	0.9	1.5
4	60	0.12	0.6	1.0
5	90	0.12	0.9	1.0
6	60	0.12	0.9	0.5
7	120	0.12	1.2	1.0
8	120	0.08	0.9	1.0
9	90	0.08	1.2	1.0
10	60	0.08	0.9	1.0
11	90	0.12	0.9	1.0
12	120	0.12	0.9	0.5
13	90	0.12	1.2	1.5
14	90	0.12	0.9	1.0
15	60	0.16	0.9	1.0
16	120	0.12	0.6	1.0
17	90	0.12	0.6	0.5
18	90	0.08	0.6	1.0
19	90	0.08	0.9	0.5
20	90	0.08	0.9	1.5
21	60	0.12	0.9	1.5
22	90	0.12	1.2	0.5
23	90	0.12	0.6	1.5
24	90	0.16	0.6	1.0
25	90	0.16	0.9	1.5
26	90	0.16	0.9	0.5
27	120	0.16	0.9	1.0

**Table 4:** Response parameter in turning of AISI 304 steel.

	Alumina			Alumina-Graphene		
S. No.	Cutting Force (N)	Surface Roughness (µm)	Temperature (°C)	Cutting Force (N)	Surface Roughness (µm)	Temperature (°C)
1	511.45	2.630	238.71	466.98	1.833	206.29
2	461.07	2.295	195.55	416.00	1.600	185.73
3	304.05	1.426	198.82	275.56	0.880	184.54
4	247.84	2.155	149.86	218.88	1.505	129.47
5	374.39	2.051	197.34	341.84	1.431	170.50
6	427.32	2.360	216.51	428.18	1.643	187.08
7	464.47	1.767	242.05	420.21	1.230	209.14
8	250.76	1.627	190.16	245.69	1.131	173.67
9	363.34	1.717	193.60	322.86	1.192	167.29
10	270.59	1.893	155.18	251.78	1.318	134.08
11	360.64	2.016	192.67	329.28	1.410	166.50
12	409.76	1.924	196.38	381.82	1.337	169.74
13	447.63	1.830	211.64	408.71	1.280	182.91
14	396.09	1.983	204.69	327.19	1.380	176.86
15	437.96	2.946	215.54	352.90	2.061	168.37
16	174.44	1.914	128.10	159.85	1.330	110.73
17	220.72	2.050	143.72	185.99	1.431	124.19
18	142.74	1.655	83.77	117.91	1.151	72.427
19	299.39	2.214	170.13	247.32	1.542	147.00
20	260.64	1.569	158.50	215.31	1.089	98.395
21	325.64	2.052	137.56	302.96	1.435	128.11
22	469.72	2.047	224.67	388.04	1.426	194.18
23	207.00	1.973	141.20	171.01	1.371	122.04
24	246.15	2.762	154.44	203.34	1.924	133.45
25	425.76	2.531	214.13	351.72	1.763	185.07
26	436.18	2.665	213.52	360.34	1.864	184.50
27	444.45	2.548	227.53	310.18	1.682	229.77

**Table 5:** ANOVA analysis of MQL machining with alumina nanofluid.

	Cutting Force (N)		Surface Roughness (μm)		Temperature (°C)	
Source	*p*-Value	% Contribution	*p*-Value	% Contribution	*p*-Value	% Contribution
Model	0.000		0.000		0.000	
Linear	0.000		0.000		0.000	
Vc	0.175	0.44779	0.000	13.28918	0.016	2.772098
fo	0.000	24.96624	0.000	62.3849	0.000	21.2578
ap	0.000	65.28413	0.574	0.104758	0.000	55.53153
np%	0.005	2.551326	0.000	7.532599	0.025	2.312737
Square	0.031		0.002		0.029	
Vc * Vc	0.651	0.04657	0.981	0.000256	0.869	0.009924
fo * fo	0.071	0.84686	0.000	7.223191	0.288	0.436902
ap * ap	0.004	2.709664	0.755	0.03176	0.003	5.074911
np%*np%	0.782	0.017195	0.484	0.163413	0.426	0.240518
2-Way Interaction	0.687		0.245		0.021	
Vc* fo	0.602	0.061974	0.562	0.111161	0.343	0.344978
Vc * ap	0.144	0.528392	0.219	0.526352	0.013	3.042387
Vc *np%	0.936	0.001433	0.406	0.231544	0.004	4.32489
fo * ap	0.381	0.179116	0.400	0.238459	0.294	0.426456
fo *np%	0.575	0.072005	0.039	1.668447	0.608	0.097931
ap *np%	0.868	0.006448	0.537	0.126273	0.660	0.072077
Error		2.592522		3.752084		4.243412
Lack-of-Fit	0.370	2.363612	0.076	3.693686	0.206	4.051467
Pure Error		0.22891		0.058398		0.191683
Total		100		100		100

**Table 6:** ANOVA analysis of MQL machining with alumina–graphene hybrid nanofluid.

	Cutting Force (N)		Surface Roughness (μm)		Temperature (°C)	
Source	*p*-Value	% Contribution	*p*-Value	% Contribution	*p*-Value	% Contribution
Model	0.000		0.000		0.000	
Linear	0.000		0.000		0.000	
V_c_	0.122	1.163	0.000	16.293	0.016	2.772098
f_o_	0.000	15.362	0.000	57.547	0.000	21.2578
a_p_	0.000	68.977	0.621	0.094	0.000	55.53153
np%	0.028	2.624	0.000	8.498	0.025	2.312737
Square	0.046		0.005		0.029	
V_c_ * V_c_	0.771	0.037	0.628	0.0906	0.869	0.009924
f_o_ * f_o_	0.020	3.0230	0.001	6.5418	0.288	0.436902
a_p_ * a_p_	0.035	2.357	0.927	0.0030	0.003	5.074911
np%*np%	0.888	0.0084	0.434	0.240	0.426	0.240518
2-Way Interaction	0.809		0.222	3.585	0.021	
V_c_* f_o_	0.562	0.149	0.283	0.462	0.343	0.344978
V_c_ * a_p_	0.324	0.443	0.276	0.478	0.013	3.042387
V_c_ *np%	0.763	0.039	0.170	0.781	0.004	4.32489
f_o_ * a_p_	0.359	0.382	0.454	0.219	0.294	0.426456
f_o_ *np%	0.710	0.060	0.062	1.552	0.608	0.097931
a_p_ *np%	0.573	0.141	0.625	0.092	0.660	0.072077
Error		5.035		4.397		4.243412
Lack-of-Fit	0.054	4.979	0.072	4.332	0.206	4.051467
Pure Error		0.055		0.064		0.191683
Total		100		100		100

**Table 7:** Analysis of MCDM techniques in alumina enriched nanofluid.

Response Parameters			Ranks by Different MCDM Techniques		
Cutting Force (N)	Surface Roughness (µm)	Temperature (°C)	MOORA	VIKOR	TOPSIS
511.45	2.630	238.71	27	27	27
461.07	2.295	195.55	19	21	21
304.05	1.426	198.82	9	10	10
247.84	2.155	149.86	8	9	7
374.39	2.051	197.34	15	14	15
427.32	2.360	216.51	21	19	22
464.47	1.767	242.05	20	22	19
250.76	1.627	190.16	7	8	8
363.34	1.717	193.60	13	12	13
270.59	1.893	155.18	6	5	6
360.64	2.016	192.67	14	13	14
409.76	1.924	196.38	16	16	16
447.63	1.830	211.64	18	18	18
396.09	1.983	204.69	17	15	17
437.96	2.946	215.54	26	26	26
174.44	1.914	128.10	2	2	2
220.72	2.050	143.72	5	6	4
142.74	1.655	83.77	1	1	1
299.39	2.214	170.13	11	11	12
260.64	1.569	158.50	4	3	5
325.64	2.052	137.56	10	7	9
469.72	2.047	224.67	22	25	20
207.00	1.973	141.20	3	4	3
246.15	2.762	154.44	12	17	11
425.76	2.531	214.13	23	20	23
436.18	2.665	213.52	24	24	24
444.45	2.548	227.53	25	23	25

**Table 8:** Analysis of MCDM techniques in alumina–graphene nanofluid.

Response Parameters with (Alumina-Graphene)			Rank by Different MCDM Techniques		
Cutting Force (N)	Surface Roughness (µm)	Temperature (°C)	MOORA	VIKOR	TOPSIS
466.98	1.833	206.29	27	27	27
416.01	1.601	185.73	24	23	25
275.56	0.881	184.54	8	12	9
218.88	1.505	129.47	6	7	6
341.84	1.431	170.50	16	15	16
428.18	1.643	187.08	26	24	26
420.21	1.231	209.14	21	22	19
245.70	1.131	173.67	9	9	8
322.86	1.193	167.29	13	11	13
251.78	1.318	134.08	7	5	7
329.28	1.410	166.50	14	13	14
381.82	1.338	169.74	17	17	17
408.71	1.281	182.91	18	20	18
327.19	1.381	176.86	15	14	15
352.90	2.061	168.37	25	26	23
159.85	1.330	110.73	3	3	3
185.99	1.431	124.19	5	6	5
117.91	1.151	72.42	1	1	1
247.32	1.542	147.00	11	10	10
215.31	1.090	98.39	2	2	2
302.96	1.436	128.11	10	8	12
388.04	1.426	194.19	19	19	20
171.01	1.371	122.04	4	4	4
203.34	1.924	133.45	12	16	11
351.72	1.763	185.07	20	18	22
360.34	1.864	184.50	23	21	24
310.18	1.683	229.77	22	25	21

**Table 9:** The optimum results through RSM, MOORA, VIKOR, and TOPSIS.

Parameters/Technique		Cutting Speed (mm/min)	Feed Rate (mm/rev)	Depth of Cut (mm)	Np%	CuttingForce (N)	Surface Roughness (μm)	Temperature (°C)
**RSM**	Alumina	86.667	0.08	0.6	1.5	101.756	1.48475	83.77
	Alumina-Graphene	110.909	0.08	0.6484	1.5	92.657	0.91186	78.766
**MOORA**	Alumina	90	0.08	0.6	1.0	142.7404	1.655947	83.77385
	Alumina-Graphene	90	0.08	0.6	1.0	117.917	1.151	72.428
**VIKOR**	Alumina	90	0.08	0.6	1.0	142.7404	1.655947	83.77385
	Alumina-Graphene	90	0.08	0.6	1.0	117.917	1.151	72.428
**TOPSIS**	Alumina	90	0.08	0.6	1.0	142.7404	1.655947	83.77385
	Alumina-Graphene	90	0.08	0.6	1.0	117.917	1.151	72.428

## Nomenclature

F_c_	Cutting force
V_c_	Cutting speed
a_p_	Depth of cut
np%	Nanofluid concentration
f_o_	Feed rate
B_i_	Assignment value
R_i_	Relation closeness
Qi	VIKOR index
u	Utility
r	Regret
s+	Separation from best solution
s−	Separation from worst solution
MQL	Minimum quality lubrication
MOORA	Multiobjective optimization on the basis of ratio analysis
VIKOR	VIšekriterijumsko KOmpromisno Rangiranje
TOPSIS	Technique for order of preferences by similarity to the ideal solution
MCDM	Multicriteria decision making
RSM	Response surface methodology

## Appendix A

**Table A1:** Analysis of variance for cutting force using alumina.

Source	DF	Adj SS	Adj MS	F-Value	*p*-Value	% Contribution	Remark
Model	14	271,912	19,422	32.21	0.000		
Linear	4	260,306	65,076	107.91	0.000		
V_c_	1	1250	1250	2.07	0.175	0.44779	
f_o_	1	69,693	69,693	115.56	0.000	24.96624	significant
a_p_	1	182,240	182,240	302.19	0.000	65.28413	significant
np%	1	7122	7122	11.81	0.005	2.551326	significant
Square	4	9236	2309	3.83	0.031		
V_c_ * V_c_	1	130	130	0.22	0.651	0.04657	
f_o_ * f_o_	1	2364	2364	3.92	0.071	0.84686	significant
a_p_ * a_p_	1	7564	7564	12.54	0.004	2.709664	significant
np%*np%	1	48	48	0.08	0.782	0.017195	
2-Way Interaction	6	2370	395	0.65	0.687		
V_c_* f_o_	1	173	173	0.29	0.602	0.061974	
V_c_ * a_p_	1	1475	1475	2.45	0.144	0.528392	
V_c_ *np%	1	4	4	0.01	0.936	0.001433	
f_o_ * a_p_	1	500	500	0.83	0.381	0.179116	
f_o_ *np%	1	201	201	0.33	0.575	0.072005	
a_p_ *np%	1	18	18	0.03	0.868	0.006448	
Error	12	7237	603			2.592522	
Lack-of-Fit	10	6598	660	2.07	0.370	2.363612	
Pure Error	2	639	319			0.22891	
Total	26	279,149				100	

**Table A2:** Analysis of variance of surface roughness using alumina.

Source	DF	Adj SS	Adj MS	F-Value	*p*-Value	% Contribution	Remark
Model	14	3.75774	0.26841	21.99	0.000		
Linear	4	3.25267	0.81317	66.61	0.000		
V_c_	1	0.51884	0.51884	42.50	0.000	13.28918	significant
f_o_	1	2.43565	2.43565	199.52	0.000	62.3849	significant
a_p_	1	0.00409	0.00409	0.33	0.574	0.104758	
np%	1	0.29409	0.29409	24.09	0.000	7.532599	significant
Square	4	0.39176	0.09794	8.02	0.002		
V_c_ * V_c_	1	0.00001	0.00001	0.00	0.981	0.000256	
f_o_ * f_o_	1	0.28201	0.28201	23.10	0.000	7.223191	significant
a_p_ * a_p_	1	0.00124	0.00124	0.10	0.755	0.03176	
np%*np%	1	0.00638	0.00638	0.52	0.484	0.163413	
2-Way Interaction	6	0.11331	0.01889	1.55	0.245		
V_c_* f_o_	1	0.00434	0.00434	0.36	0.562	0.111161	
V_c_ * a_p_	1	0.02055	0.02055	1.68	0.219	0.526352	
V_c_ *np%	1	0.00904	0.00904	0.74	0.406	0.231544	
f_o_ * a_p_	1	0.00931	0.00931	0.76	0.400	0.238459	
f_o_ *np%	1	0.06514	0.06514	5.34	0.039	1.668447	
a_p_ *np%	1	0.00493	0.00493	0.40	0.537	0.126273	
Error	12	0.14649	0.01221			3.752084	
Lack-of-Fit	10	0.14421	0.01442	12.63	0.076	3.693686	
Pure Error	2	0.00228	0.00114			0.058398	
Total	26	3.90423				100	

**Table A3:** Analysis of variance of temperature using alumina.

Source	DF	Adj SS	Adj MS	F-Value	*p*-Value	% Contribution	Remark
Model	14	36,667.4	2619.1	19.34	0.000		
Linear	4	31,351.5	7837.9	57.88	0.000		
V_c_	1	1061.5	1061.5	7.84	0.016	2.772098	significant
f_o_	1	8140.1	8140.1	60.12	0.000	21.2578	significant
a_p_	1	21,264.3	21,264.3	157.04	0.000	55.53153	significant
np%	1	885.6	885.6	6.54	0.025	2.312737	significant
Square	4	2134.3	533.6	3.94	0.029		
V_c_ * V_c_	1	3.8	3.8	0.03	0.869	0.009924	
f_o_ * f_o_	1	167.3	167.3	1.24	0.288	0.436902	
a_p_ * a_p_	1	1943.3	1943.3	14.35	0.003	5.074911	significant
np%*np%	1	92.1	92.1	0.68	0.426	0.240518	
2-Way Interaction	6	3181.6	530.3	3.92	0.021		
V_c_* f_o_	1	132.1	132.1	0.98	0.343	0.344978	
V_c_ * a_p_	1	1165.0	1165.0	8.60	0.013	3.042387	significant
V_c_ *np%	1	1656.1	1656.1	12.23	0.004	4.32489	significant
f_o_ * a_p_	1	163.3	163.3	1.21	0.294	0.426456	
f_o_ *np%	1	37.5	37.5	0.28	0.608	0.097931	
a_p_ *np%	1	27.6	27.6	0.20	0.660	0.072077	
Error	12	1624.9	135.4			4.243412	
Lack-of-Fit	10	1551.4	155.1	4.23	0.206	4.051467	
Pure Error	2	73.4	36.7			0.191683	
Total	26	38,292.3				100	

**Table A4:** Analysis of variance for force using alumina–graphene.

Source	DF	Adj SS	Adj MS	F-Value	*p*-Value	% Contribution	Remark
Model	14	214,022	15,287	16.17	0.000		
Linear	4	198,614	49,654	52.51	0.000		
V_c_	1	2623	2623	2.77	0.122	1.163	
f_o_	1	34,622	34,622	36.61	0.000	15.362	significant
a_p_	1	155,455	155,455	164.39	0.000	68.977	significant
np%	1	5915	5915	6.25	0.028	2.624	significant
Square	4	12,667	3167	3.35	0.046		
V_c_ * V_c_	1	84	84	0.09	0.771	0.037	
f_o_ * f_o_	1	6813	6813	7.20	0.020	3.0230	significant
a_p_ * a_p_	1	5312	5312	5.62	0.035	2.357	significant
np%*np%	1	19	19	0.02	0.888	0.0084	
2-Way Interaction	6	2741	457	0.48	0.809		
V_c_* f_o_	1	336	336	0.35	0.562	0.149	
V_c_ * a_p_	1	999	999	1.06	0.324	0.443	
V_c_ *np%	1	90	90	0.10	0.763	0.039	
f_o_ * a_p_	1	861	861	0.91	0.359	0.382	
f_o_ *np%	1	137	137	0.14	0.710	0.060	
a_p_ *np%	1	318	318	0.34	0.573	0.141	
Error	12	11,348	946			5.035	
Lack-of-Fit	10	11,222	1122	17.88	0.054	4.979	
Pure Error	2	126	63			0.055	
Total	26	225,370				100	

**Table A5:** Analysis of variance of surface roughness using alumina–graphene.

Source	DF	Adj SS	Adj MS	F-Value	*p*-Value	% Contribution	Remark
Model	14	1.89893	0.13564	18.63	0.000		
Linear	4	1.63737	0.40934	56.24	0.000		
V_c_	1	0.32364	0.32364	44.46	0.000	16.293	significant
f_o_	1	1.14306	1.14306	157.04	0.000	57.547	significant
a_p_	1	0.00188	0.00188	0.26	0.621	0.094	
np%	1	0.16880	0.16880	23.19	0.000	8.498	significant
Square	4	0.19034	0.04758	6.54	0.005		
V_c_ * V_c_	1	0.00180	0.00180	0.25	0.628	0.0906	
f_o_ * f_o_	1	0.12994	0.12994	17.85	0.001	6.5418	significant
a_p_ * a_p_	1	0.00006	0.00006	0.01	0.927	0.0030	
np%*np%	1	0.00477	0.00477	0.66	0.434	0.240	
2-Way Interaction	6	0.07122	0.01187	1.63	0.222	3.585	
V_c_* f_o_	1	0.00918	0.00918	1.26	0.283	0.462	
V_c_ * a_p_	1	0.00950	0.00950	1.30	0.276	0.478	
V_c_ *np%	1	0.01552	0.01552	2.13	0.170	0.781	
f_o_ * a_p_	1	0.00436	0.00436	0.60	0.454	0.219	
f_o_ *np%	1	0.03084	0.03084	4.24	0.062	1.552	
a_p_ *np%	1	0.00183	0.00183	0.25	0.625	0.092	
Error	12	0.08734	0.00728			4.397	
Lack-of-Fit	10	0.08606	0.00861	13.34	0.072	4.332	
Pure Error	2	0.00129	0.00064			0.064	
Total	26	1.98628				100	

**Table A6:** Analysis of variance of temperature using alumina–graphene.

Source	DF	Adj SS	Adj MS	F-Value	*p*-Value	% Contribution	Remark
Model	14	32,997.8	2357.0	12.40	0.000		
Linear	4	28,041.7	7010.4	36.88	0.000		
V_c_	1	1746.0	1746.0	9.18	0.010	4.949	significant
f_o_	1	8247.7	8247.7	43.39	0.000	23.378	significant
a_p_	1	17,118.4	17,118.4	90.05	0.000	48.522	significant
np%	1	929.6	929.6	4.89	0.047	2.6349	significant
Square	4	2285.2	571.3	3.01	0.062		
V_c_ * V_c_	1	201.8	201.8	1.06	0.323	0.572	
f_o_ * f_o_	1	309.2	309.2	1.63	0.226	0.876	
a_p_ * a_p_	1	1267.8	1267.8	6.67	0.024	3.593	significant
np%*np%	1	238.0	238.0	1.25	0.285	0.674	
2-Way Interaction	6	2671.0	445.2	2.34	0.099		
V_c_* f_o_	1	118.9	118.9	0.63	0.444	0.337	
V_c_ * a_p_	1	444.5	444.5	2.34	0.152	1.259	
V_c_ *np%	1	1360.8	1360.8	7.16	0.020	3.857	significant
f_o_ * a_p_	1	121.3	121.3	0.64	0.440	0.343	
f_o_ *np%	1	604.7	604.7	3.18	0.100	1.7140	
a_p_ *np%	1	20.8	20.8	0.11	0.747	0.0589	
Error	12	2281.2	190.1			6.4661	
Lack-of-Fit	10	2226.7	222.7	8.16	0.114	6.3116	
Pure Error	2	54.6	27.3			0.1547	
Total	26	35,279.0				100	

**Table A7:** MOORA analysis for alumina.

Decision Matrix			Normalizing Matrix				
Cutting Force (N)	Surface Rough Ness (µm)	Temperature (°C)				B	Rank
511.4568	2.63064	238.717	0.2719	0.2376	0.2433	−0.3764	27
461.075	2.29599	195.552	0.2451	0.2074	0.1993	−0.3259	19
304.0594	1.426832	198.8272	0.1617	0.1289	0.2026	−0.2466	9
247.841	2.15581	149.8645	0.1318	0.1947	0.1527	−0.2396	8
374.3974	2.051186	197.3411	0.1990	0.1852	0.2011	−0.2927	15
427.3259	2.360216	216.5133	0.2272	0.2132	0.2207	−0.3305	21
464.4795	1.767456	242.0562	0.2469	0.1596	0.2467	−0.3266	20
250.7642	1.627584	190.1616	0.1333	0.1470	0.1938	−0.2371	7
363.342	1.717272	193.6079	0.1932	0.1551	0.1973	−0.2728	13
270.5931	1.893312	155.181	0.1439	0.1710	0.1582	−0.2365	6
360.6416	2.016965	192.6746	0.1917	0.1822	0.1964	−0.2851	14
409.7601	1.924486	196.3889	0.2178	0.1738	0.2002	−0.2959	16
447.6368	1.830473	211.6454	0.2380	0.1653	0.2157	−0.3095	18
396.0915	1.983618	204.6936	0.2106	0.1791	0.2086	−0.2992	17
437.9675	2.946243	215.5425	0.2328	0.2661	0.2197	−0.3593	26
174.4423	1.914002	128.1041	0.0927	0.1729	0.1306	−0.1981	2
220.7251	2.050069	143.7265	0.1173	0.1851	0.1465	−0.2245	5
142.7404	1.655947	83.77385	0.0759	0.1495	0.0854	−0.1554	1
299.3917	2.214356	170.1335	0.1592	0.2000	0.1734	−0.2663	11
260.6497	1.569603	158.5022	0.1386	0.1418	0.1615	−0.2209	4
325.648	2.052732	137.5602	0.1731	0.1854	0.1402	−0.2494	10
469.7263	2.047881	224.6752	0.2497	0.1849	0.2290	−0.3318	22
207.0041	1.973061	141.2001	0.1101	0.1782	0.1439	−0.2161	3
246.1514	2.76224	154.44	0.1309	0.2495	0.1574	−0.2689	12
425.7669	2.531105	214.1387	0.2264	0.2286	0.2182	−0.3366	23
436.1839	2.665395	213.5229	0.2319	0.2407	0.2176	−0.3451	24
444.4571	2.54873	227.5397	0.2363	0.2302	0.2319	−0.3492	25

**Table A8:** MOORA analysis for alumina–graphene.

Decision Matrix			Normalizing Matrix				
Cutting Force (N)	Surface Rough Ness (µm)	Temperature (°C)				B	Rank
466.982	1.833	206.295	0.2833	0.2386	0.2409	−0.3814	27
416.010	1.601	185.731	0.2524	0.2083	0.2168	−0.3388	24
275.566	0.881	184.549	0.1672	0.1146	0.2155	−0.2486	8
218.882	1.505	129.479	0.1328	0.1959	0.1512	−0.2399	6
341.841	1.431	170.509	0.2074	0.1862	0.1991	−0.2964	16
428.187	1.643	187.083	0.2598	0.2139	0.2184	−0.3460	26
420.214	1.231	209.147	0.2549	0.1602	0.2442	−0.3296	21
245.700	1.131	173.671	0.1491	0.1472	0.2028	−0.2495	9
322.866	1.193	167.294	0.1959	0.1552	0.1953	−0.2732	13
251.789	1.318	134.084	0.1528	0.1716	0.1565	−0.2404	7
329.283	1.410	166.504	0.1998	0.1835	0.1944	−0.2889	14
381.823	1.338	169.741	0.2316	0.1741	0.1982	−0.3020	17
408.718	1.281	182.915	0.2480	0.1667	0.2136	−0.3141	18
327.195	1.381	176.862	0.1985	0.1797	0.2065	−0.2923	15
352.906	2.061	168.371	0.2141	0.2683	0.1966	−0.3395	25
159.859	1.330	110.731	0.0970	0.1731	0.1293	−0.1997	3
185.999	1.431	124.199	0.1128	0.1863	0.1450	−0.2221	5
117.917	1.151	72.428	0.0715	0.1498	0.0846	−0.1530	1
247.324	1.542	147.002	0.1500	0.2007	0.1716	−0.2612	11
215.319	1.090	98.396	0.1306	0.1418	0.1149	−0.1937	2
302.967	1.436	128.114	0.1838	0.1868	0.1496	−0.2601	10
388.041	1.426	194.190	0.2354	0.1856	0.2267	−0.3239	19
171.010	1.371	122.044	0.1037	0.1785	0.1425	−0.2124	4
203.345	1.924	133.458	0.1234	0.2504	0.1558	−0.2648	12
351.721	1.763	185.077	0.2134	0.2294	0.2161	−0.3295	20
360.343	1.864	184.502	0.2186	0.2426	0.2154	−0.3383	23
310.181	1.683	229.770	0.1882	0.2190	0.2683	−0.3377	22

**Table A9:** VIKOR analysis for alumina.

Decision Matrix			Normalizing Matrix						
Cutting Force (N)	Surface Rough Ness (µm)	Temperature (°C)				u	r	Q	Rank
511.4568	2.63064	238.717	0.2719	0.2376	0.2433	−0.5797	−0.1932	1.0000	27
461.075	2.29599	195.552	0.2451	0.2074	0.1993	−0.5798	−0.1932	0.7763	21
304.0594	1.426832	198.8272	0.1617	0.1289	0.2026	−0.5800	−0.1933	0.4232	10
247.841	2.15581	149.8645	0.1318	0.1947	0.1527	−0.5800	−0.1933	0.3750	9
374.3974	2.051186	197.3411	0.1990	0.1852	0.2011	−0.5799	−0.1933	0.5214	14
427.3259	2.360216	216.5133	0.2272	0.2132	0.2207	−0.5798	−0.1933	0.7134	19
464.4795	1.767456	242.0562	0.2469	0.1596	0.2467	−0.5798	−0.1932	0.7853	22
250.7642	1.627584	190.1616	0.1333	0.1470	0.1938	−0.5800	−0.1933	0.3656	8
363.342	1.717272	193.6079	0.1932	0.1551	0.1973	−0.5800	−0.1933	0.4608	12
270.5931	1.893312	155.181	0.1439	0.1710	0.1582	−0.5801	−0.1933	0.2711	5
360.6416	2.016965	192.6746	0.1917	0.1822	0.1964	−0.5799	−0.1933	0.4848	13
409.7601	1.924486	196.3889	0.2178	0.1738	0.2002	−0.5799	−0.1933	0.5970	16
447.6368	1.830473	211.6454	0.2380	0.1653	0.2157	−0.5799	−0.1932	0.7100	18
396.0915	1.983618	204.6936	0.2106	0.1791	0.2086	−0.5799	−0.1933	0.5747	15
437.9675	2.946243	215.5425	0.2328	0.2661	0.2197	−0.5797	−0.1932	0.9375	26
174.4423	1.914002	128.1041	0.0927	0.1729	0.1306	−0.5802	−0.1933	0.1918	2
220.7251	2.050069	143.7265	0.1173	0.1851	0.1465	−0.5801	−0.1933	0.3017	6
142.7404	1.655947	83.77385	0.0759	0.1495	0.0854	−0.5803	−0.1934	0.0000	1
299.3917	2.214356	170.1335	0.1592	0.2000	0.1734	−0.5800	−0.1933	0.4569	11
260.6497	1.569603	158.5022	0.1386	0.1418	0.1615	−0.5801	−0.1933	0.1972	3
325.648	2.052732	137.5602	0.1731	0.1854	0.1402	−0.5800	−0.1933	0.3590	7
469.7263	2.047881	224.6752	0.2497	0.1849	0.2290	−0.5798	−0.1932	0.8085	25
207.0041	1.973061	141.2001	0.1101	0.1782	0.1439	−0.5801	−0.1933	0.2543	4
246.1514	2.76224	154.44	0.1309	0.2495	0.1574	−0.5800	−0.1932	0.6650	17
425.7669	2.531105	214.1387	0.2264	0.2286	0.2182	−0.5798	−0.1933	0.7329	20
436.1839	2.665395	213.5229	0.2319	0.2407	0.2176	−0.5798	−0.1932	0.8017	24
444.4571	2.54873	227.5397	0.2363	0.2302	0.2319	−0.5797	−0.1932	0.7929	23

**Table A10:** VIKOR analysis for alumina–graphene.

Decision Matrix			Normalizing Matrix						
Cutting Force (N)	Surface Rough Ness (µm)	Temperature (°C)				u	r	Q	Rank
466.982	1.833	206.295	0.2833	0.2386	0.2409	−0.5797	−0.1932	1.0000	27
416.010	1.601	185.731	0.2524	0.2083	0.2168	−0.5798	−0.1932	0.7975	23
275.566	0.881	184.549	0.1672	0.1146	0.2155	−0.5800	−0.1933	0.4698	12
218.882	1.505	129.479	0.1328	0.1959	0.1512	−0.5800	−0.1933	0.3816	7
341.841	1.431	170.509	0.2074	0.1862	0.1991	−0.5799	−0.1933	0.5456	15
428.187	1.643	187.083	0.2598	0.2139	0.2184	−0.5798	−0.1932	0.8395	24
420.214	1.231	209.147	0.2549	0.1602	0.2442	−0.5798	−0.1932	0.7865	22
245.700	1.131	173.671	0.1491	0.1472	0.2028	−0.5800	−0.1933	0.4268	9
322.866	1.193	167.294	0.1959	0.1552	0.1953	−0.5800	−0.1933	0.4543	11
251.789	1.318	134.084	0.1528	0.1716	0.1565	−0.5800	−0.1933	0.2966	5
329.283	1.410	166.504	0.1998	0.1835	0.1944	−0.5799	−0.1933	0.5023	13
381.823	1.338	169.741	0.2316	0.1741	0.1982	−0.5799	−0.1932	0.6436	17
408.718	1.281	182.915	0.2480	0.1667	0.2136	−0.5798	−0.1932	0.7278	20
327.195	1.381	176.862	0.1985	0.1797	0.2065	−0.5799	−0.1933	0.5337	14
352.906	2.061	168.371	0.2141	0.2683	0.1966	−0.5798	−0.1932	0.8551	26
159.859	1.330	110.731	0.0970	0.1731	0.1293	−0.5802	−0.1933	0.2131	3
185.999	1.431	124.199	0.1128	0.1863	0.1450	−0.5801	−0.1933	0.3083	6
117.917	1.151	72.428	0.0715	0.1498	0.0846	−0.5803	−0.1934	0.0000	1
247.324	1.542	147.002	0.1500	0.2007	0.1716	−0.5800	−0.1933	0.4450	10
215.319	1.090	98.396	0.1306	0.1418	0.1149	−0.5802	−0.1934	0.0891	2
302.967	1.436	128.114	0.1838	0.1868	0.1496	−0.5800	−0.1933	0.3937	8
388.041	1.426	194.190	0.2354	0.1856	0.2267	−0.5798	−0.1932	0.7049	19
171.010	1.371	122.044	0.1037	0.1785	0.1425	−0.5801	−0.1933	0.2597	4
203.345	1.924	133.458	0.1234	0.2504	0.1558	−0.5800	−0.1932	0.6285	16
351.721	1.763	185.077	0.2134	0.2294	0.2161	−0.5798	−0.1933	0.6960	18
360.343	1.864	184.502	0.2186	0.2426	0.2154	−0.5798	−0.1932	0.7620	21
310.181	1.683	229.770	0.1882	0.2190	0.2683	−0.5798	−0.1932	0.8513	25

**Table A11:** TOPSIS analysis for alumina.

Decision Matrix			Normalizing Matrix						
Cutting Force (N)	Surface Rough Ness (µm)	Temperature (°C)				S+	S−	Ri	Rank
511.4568	2.63064	238.717	0.2719	0.2376	0.2433	0.1371	0.0144	0.095	27
461.075	2.29599	195.552	0.2451	0.2074	0.1993	0.1093	0.0400	0.268	21
304.0594	1.426832	198.8272	0.1617	0.1289	0.2026	0.0726	0.0907	0.555	10
247.841	2.15581	149.8645	0.1318	0.1947	0.1527	0.0548	0.0916	0.626	7
374.3974	2.051186	197.3411	0.1990	0.1852	0.2011	0.0891	0.0590	0.398	15
427.3259	2.360216	216.5133	0.2272	0.2132	0.2207	0.1099	0.0370	0.252	22
464.4795	1.767456	242.0562	0.2469	0.1596	0.2467	0.1186	0.0547	0.316	19
250.7642	1.627584	190.1616	0.1333	0.1470	0.1938	0.0620	0.0951	0.605	8
363.342	1.717272	193.6079	0.1932	0.1551	0.1973	0.0821	0.0724	0.468	13
270.5931	1.893312	155.181	0.1439	0.1710	0.1582	0.0541	0.0912	0.628	6
360.6416	2.016965	192.6746	0.1917	0.1822	0.1964	0.0845	0.0633	0.428	14
409.7601	1.924486	196.3889	0.2178	0.1738	0.2002	0.0940	0.0583	0.383	16
447.6368	1.830473	211.6454	0.2380	0.1653	0.2157	0.1056	0.0554	0.344	18
396.0915	1.983618	204.6936	0.2106	0.1791	0.2086	0.0947	0.0565	0.374	17
437.9675	2.946243	215.5425	0.2328	0.2661	0.2197	0.1240	0.0238	0.161	26
174.4423	1.914002	128.1041	0.0927	0.1729	0.1306	0.0326	0.1165	0.781	2
220.7251	2.050069	143.7265	0.1173	0.1851	0.1465	0.0464	0.1006	0.684	4
142.7404	1.655947	83.77385	0.0759	0.1495	0.0854	0.0103	0.1397	0.931	1
299.3917	2.214356	170.1335	0.1592	0.2000	0.1734	0.0703	0.0749	0.516	12
260.6497	1.569603	158.5022	0.1386	0.1418	0.1615	0.0497	0.1006	0.669	5
325.648	2.052732	137.5602	0.1731	0.1854	0.1402	0.0626	0.0831	0.570	9
469.7263	2.047881	224.6752	0.2497	0.1849	0.2290	0.1162	0.0430	0.270	20
207.0041	1.973061	141.2001	0.1101	0.1782	0.1439	0.0419	0.1055	0.716	3
246.1514	2.76224	154.44	0.1309	0.2495	0.1574	0.0754	0.0839	0.527	11
425.7669	2.531105	214.1387	0.2264	0.2286	0.2182	0.1121	0.0328	0.226	23
436.1839	2.665395	213.5229	0.2319	0.2407	0.2176	0.1165	0.0278	0.193	24
444.4571	2.54873	227.5397	0.2363	0.2302	0.2319	0.1199	0.0263	0.180	25

**Table A12:** TOPSIS analysis for alumina–graphene.

Decision Matrix			Normalizing Matrix						
Cutting Force (N)	Surface Rough Ness (µm)	Temperature (°C)				S+	S−	Ri	Rank
466.982	1.833	206.295	0.2833	0.2386	0.2409	0.1455	0.0202	0.122	27
416.010	1.601	185.731	0.2524	0.2083	0.2168	0.1214	0.0424	0.259	25
275.566	0.881	184.549	0.1672	0.1146	0.2155	0.0811	0.0998	0.552	9
218.882	1.505	129.479	0.1328	0.1959	0.1512	0.0608	0.1020	0.626	6
341.841	1.431	170.509	0.2074	0.1862	0.1991	0.0958	0.0657	0.407	16
428.187	1.643	187.083	0.2598	0.2139	0.2184	0.1257	0.0387	0.235	26
420.214	1.231	209.147	0.2549	0.1602	0.2442	0.1237	0.0572	0.316	19
245.700	1.131	173.671	0.1491	0.1472	0.2028	0.0725	0.0961	0.570	8
322.866	1.193	167.294	0.1959	0.1552	0.1953	0.0857	0.0802	0.484	13
251.789	1.318	134.084	0.1528	0.1716	0.1565	0.0613	0.0986	0.617	7
329.283	1.410	166.504	0.1998	0.1835	0.1944	0.0912	0.0700	0.434	14
381.823	1.338	169.741	0.2316	0.1741	0.1982	0.1026	0.0641	0.385	17
408.718	1.281	182.915	0.2480	0.1667	0.2136	0.1123	0.0603	0.349	18
327.195	1.381	176.862	0.1985	0.1797	0.2065	0.0938	0.0687	0.423	15
352.906	2.061	168.371	0.2141	0.2683	0.1966	0.1188	0.0498	0.295	23
159.859	1.330	110.731	0.0970	0.1731	0.1293	0.0390	0.1256	0.763	3
185.999	1.431	124.199	0.1128	0.1863	0.1450	0.0512	0.1129	0.688	5
117.917	1.151	72.428	0.0715	0.1498	0.0846	0.0176	0.1522	0.896	1
247.324	1.542	147.002	0.1500	0.2007	0.1716	0.0727	0.0890	0.550	10
215.319	1.090	98.396	0.1306	0.1418	0.1149	0.0359	0.1253	0.777	2
302.967	1.436	128.114	0.1838	0.1868	0.1496	0.0742	0.0875	0.541	12
388.041	1.426	194.190	0.2354	0.1856	0.2267	0.1141	0.0521	0.313	20
171.010	1.371	122.044	0.1037	0.1785	0.1425	0.0460	0.1184	0.720	4
203.345	1.924	133.458	0.1234	0.2504	0.1558	0.0809	0.0982	0.548	11
351.721	1.763	185.077	0.2134	0.2294	0.2161	0.1125	0.0477	0.298	22
360.343	1.864	184.502	0.2186	0.2426	0.2154	0.1174	0.0437	0.271	24
310.181	1.683	229.770	0.1882	0.2190	0.2683	0.1207	0.0536	0.307	21
